# Solute carrier-related signature for assessing prognosis and immunity in patients with clear-cell renal cell carcinoma

**DOI:** 10.32604/or.2023.028051

**Published:** 2023-04-10

**Authors:** WEI BAO, QIANGUANG HAN, XIAO GUAN, ZIJIE WANG, MIN GU

**Affiliations:** 1 Department of Urology, The Second Affiliated Hospital of Nanjing Medical University, Nanjing, China; 2 Department of Urology, The First Affiliated Hospital of Nanjing Medical University, Nanjing, China; 3 Department of General Surgery, The Second Affiliated Hospital of Nanjing Medical University, Nanjing, China

**Keywords:** Clear cell renal cell carcinoma, Solute carrier, Bioinformatics, Metabolic reprogramming, Immune microenvironment

## Abstract

**Background:**

Clear-cell renal cell carcinoma (ccRCC) is the most common malignant kidney cancer. However, the tumor microenvironment and crosstalk involved in metabolic reprogramming in ccRCC are not well-understood.

**Methods:**

We used The Cancer Genome Atlas to obtain ccRCC transcriptome data and clinical information. The E-MTAB-1980 cohort was used for external validation. The GENECARDS database contains the first 100 solute carrier (SLC)-related genes. The predictive value of SLC-related genes for ccRCC prognosis and treatment was assessed using univariate Cox regression analysis. An SLC-related predictive signature was developed through Lasso regression analysis and used to determine the risk profiles of patients with ccRCC. Patients in each cohort were separated into high- and low-risk groups based on their risk scores. The clinical importance of the signature was assessed through survival, immune microenvironment, drug sensitivity, and nomogram analyses using R software.

**Results:**

*SLC25A23*, *SLC25A42*, *SLC5A1*, *SLC3A1*, *SLC25A37*, *SLC5A6*, *SLCO5A1*, and *SCP2* comprised the signatures of the eight SLC-related genes. Patients with ccRCC were separated into high- and low-risk groups based on the risk value in the training and validation cohorts; the high-risk group had a significantly worse prognosis (*p* < 0.001). The risk score was an independent predictive indicator of ccRCC in the two cohorts according to univariate and multivariate Cox regression (*p* < 0.05). Analysis of the immune microenvironment showed that immune cell infiltration and immune checkpoint gene expression differed between the two groups (*p* < 0.05). Drug sensitivity analysis showed that compared to the low-risk group, the high-risk group was more sensitive to sunitinib, nilotinib, JNK-inhibitor-VIII, dasatinib, bosutinib, and bortezomib (*p* < 0.001). Survival analysis and receiver operating characteristic curves were validated using the E-MTAB-1980 cohort.

**Conclusions:**

SLC-related genes have predictive relevance in ccRCC and play roles in the immunological milieu. Our results provide insight into metabolic reprogramming in ccRCC and identify promising treatment targets for ccRCC.

## Introduction

As the most common subtype of renal cancer, clear-cell renal cell carcinoma (ccRCC) is diagnosed in approximately 400,000 patients each year worldwide; around one-third of these cases are metastatic, posing a heavy burden on global health [1–3]. High levels of metabolic reprogramming and immune cell infiltration as well as active angiogenesis are thought to drive ccRCC growth and progression [4–6]. Early ccRCC can be resected through surgical treatment [7]. However, for some patients with unresectable ccRCC or with metastatic ccRCC, treatment options remain limited [8]. ccRCC is typically insensitive to cytotoxic therapy [9]. Tyrosine kinase inhibitors, which target the vascular endothelial growth factor receptor pathway, have been developed to treat ccRCC based on their angiogenic activity [10–12]. Although immunotherapeutic drugs, such as immune checkpoint inhibitors, are effective against ccRCC [13], some patients develop drug resistance, resulting in poor treatment efficiency, metastasis, recurrence, and death [14]. Thus, there is an urgent need to identify new prognostic biomarkers for ccRCC to guide diagnosis and treatment.

Solute carrier (SLC) transporters mediate secondary active transport of a variety of substances through electrochemical gradients. Their substrates include numerous metabolites, metal ions, amino acids, nucleotides, and vitamins [15–17]. The SLC transporter family consists of 52 subgroups with more than 400 members [18]. Changes in SLC transporters in cancer are considered as potential targets for cancer therapy because of their critical roles in maintaining the normal metabolic functions of cells [19]. Metabolism is highly active in cancer cells [20]. SLC transporters are often dysregulated in multiple cancer types to meet the high metabolic needs of cancer cells [21]. Understanding the metabolic patterns of ccRCC may provide insights into precise treatment strategies. However, the roles of SLC transporters in ccRCC remain unclear.

Bioinformatics is an important tool for studying biological phenomena [22] through the integration of biological and computational sciences [23]. Bioinformatics methods can be used understand disease mechanisms using discrete data. Transcriptome sequencing from The Cancer Genome Atlas (TCGA) database has been widely employed in cancer research to identify biomarkers and understand their roles in the tumor microenvironment.

**FIGURE 1:**
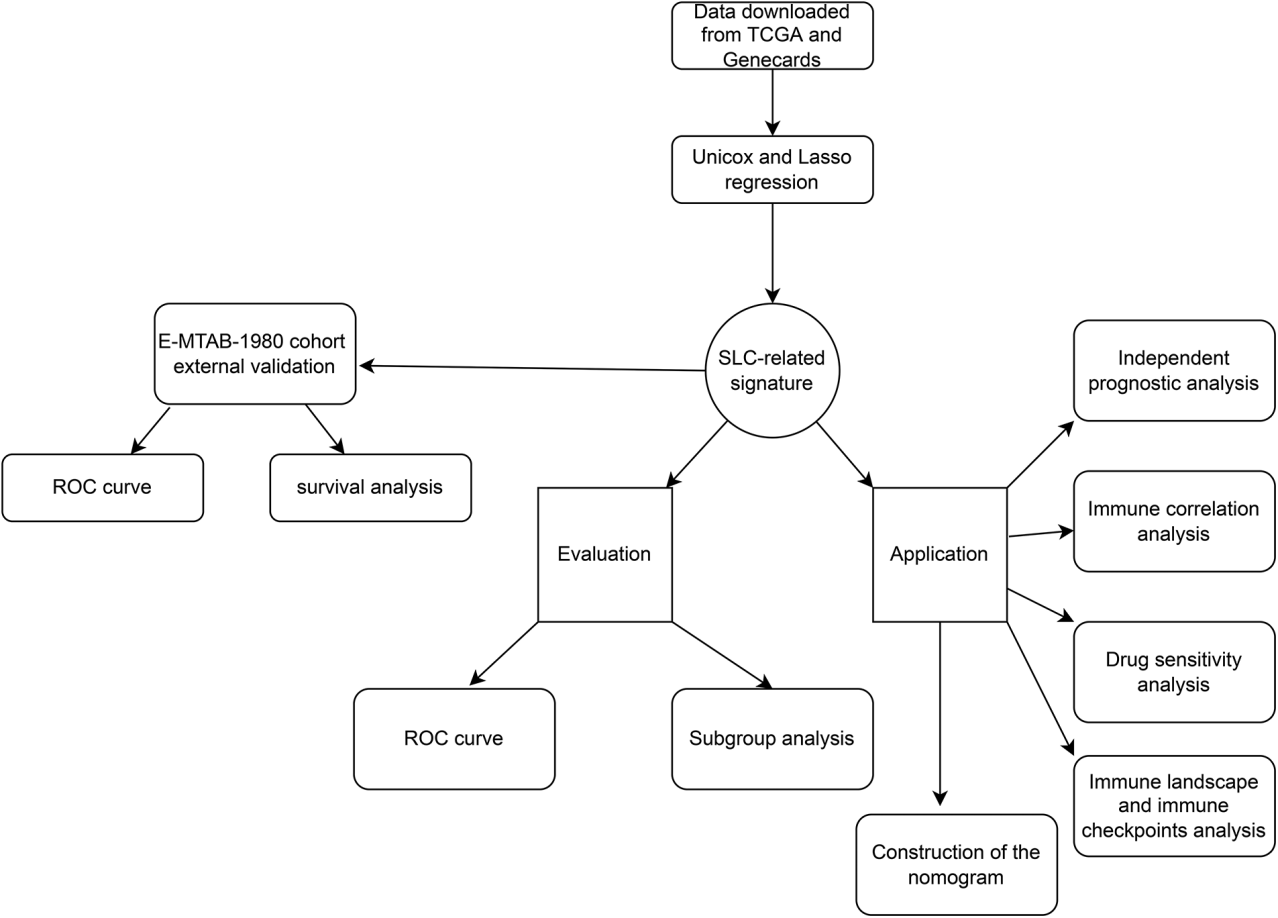
The flowchart of this work.

Here, we explored the roles of SLC-related genes in ccRCC through bioinformatics analysis. Expression, survival, and immune microenvironment analyses were conducted to construct an SLC-related prognostic signature. By using this signature, patients with ccRCC can be prognostically stratified, which has implications for treatment. A flowchart of this study is shown in Fig. 1.

## Materials and Methods

### Data downloading and preprocessing

We downloaded ccRCC transcriptome data from TCGA (https://portal.gdc.cancer.gov/). The data were searched using the terms “kidney” (Primary Site), “TCGA” (Program Name), “TCGA-KIRC” (Project Name), “transcriptome profiling” (Data Category),“Gene Expression Quantification” (Data Type), and “RNA-Seq” (Experimental Strategy). The workflow used was fragments per kilobase of exon per million mapped reads. Finally, 539 tumor samples and 72 normal samples were included in the study. The clinical files for the project were also downloaded (Suppl. Table 1). The E-MTAB-1980 cohort used for external validation was downloaded from the Array Express database (https://ebi.ac.uk/biostudies/arrayexpress/studies/E-MTAB-1980). This cohort contained the transcriptome profiles and corresponding clinical information for 101 patients with ccRCC from Japan. We processed the data using Perl language to access and transform the fpkm matrix file.

### SLC-related gene identification and univariate Cox regression

SLC-related genes were identified using the GENECARDS website (https://www.genecards.com/). The top 100 related genes ranked by their relevance scores on GENECARDS were selected for further analysis. Univariate Cox analysis was conducted using the “survival” package in R software (version 4.1.4; The R Project for Statistical Computing, Vienna, Austria).

### Lasso regression and gene signature construction

Lasso regression was performed using the R software package “glmnet”. The samples in TCGA database were randomly divided into two cohorts: a training cohort and an internal validation cohort. Receiver operating characteristic (ROC) plots were constructed, and the area under the ROC curve (AUC) was calculated to evaluate the model’s predictive ability.

### Univariate and multivariate cox regression

The survival package was employed to calculate the survival probability of the high- and low-risk groups, and a log-rank test was performed to verify the survival differences between the two groups. Multivariate and univariate Cox analyses were performed based on the risk score and clinical information.

### Immune infiltration analysis and immune checkpoint analysis

To explore the internal correlation between our risk model and the tumor infiltration level, we employed the immune infiltration heatmap and correlation map for data visualization. CIBERSORT and XCELL were used to evaluate tumor infiltration. CIBERSORT, developed by Newman et al., is used to estimate the abundance of member cell types in a mixed cell population based on gene expression data [24]. XCELL is used to identify heterogeneous tissue cellular landscapes.

### Drug sensitivity analysis and nomogram construction

The “pRRophetic” package was used to predict the drug sensitivity of different bulk-seq samples according to the transcriptome data. We employed “DynNom” R software to create a nomogram incorporating patient risk scores and clinical data to further analyze the prognosis of patients with ccRCC.

## Results

### Univariate Cox and Lasso regression to construct SLC-related prognostic signature

**FIGURE 2:**
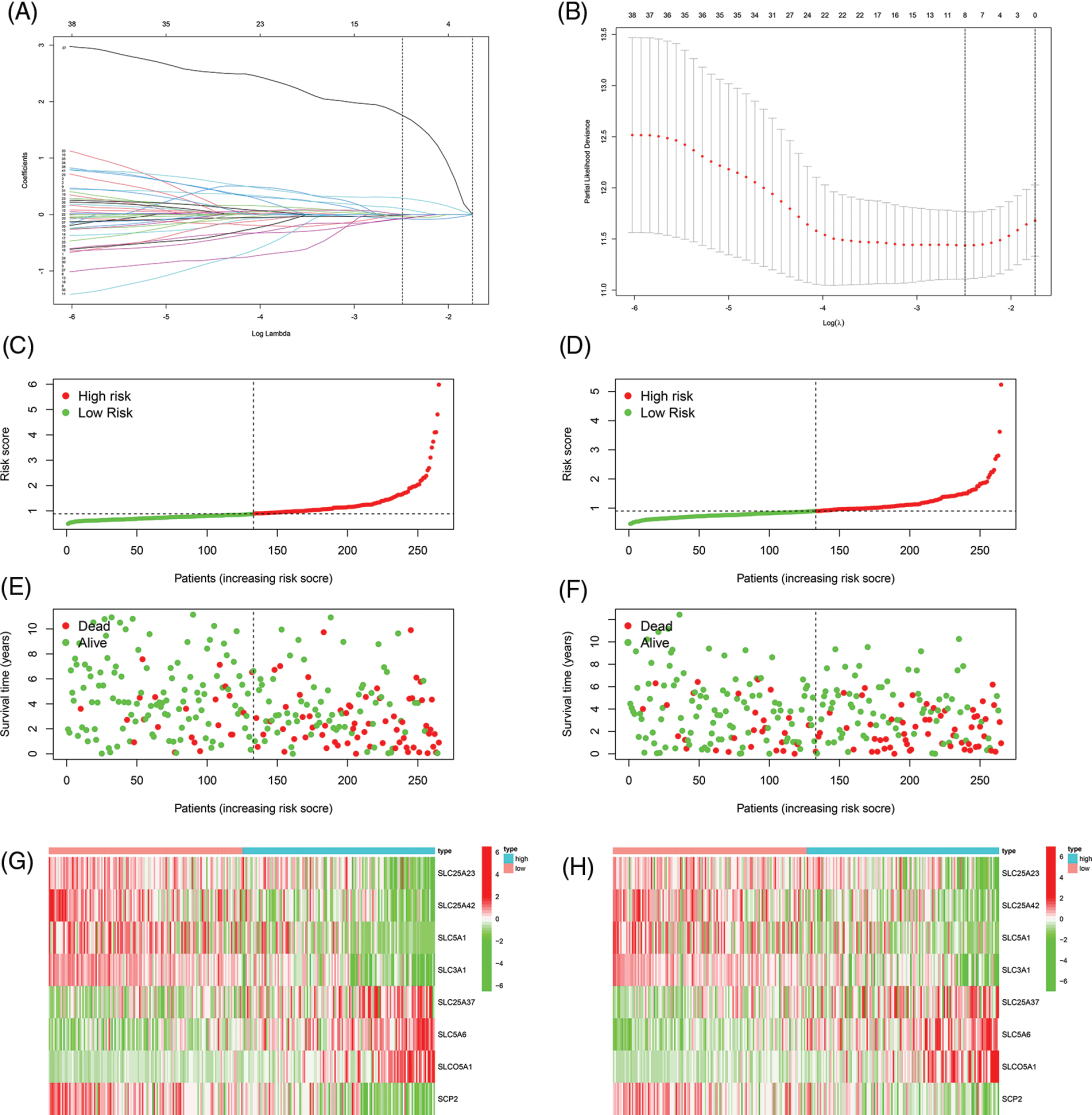
Constructing of the prognostic signature. (A, B) Lasso regression to construct prognostic signature, the curve converged when Lamda was eight. (C) Risk score of training cohort. (D) Risk score of internal validation cohort. (E) The correlation between risk score and survival status of patients in training cohort. (F) The correlation between risk score and survival status of patients in internal validation cohort. (G) Heat map of eight model genes expression in training cohort. (H) Heat map of eight model genes expression in internal validation cohort.

First, we performed univariate Cox regression of the 100 SLC genes in the training cohort and identified 41 genes with prognostic significance (Suppl. Table 2). Subsequently, Lasso regression was performed on these 41 genes, and a prognostic signature was developed (Figs. 2A and 2B). Risk score = SLC25A23 × (−0.0656830321767743) + SLC25A42 × (−0.0766650501411658) + SLC5A1 × (−0.00980404711094937) + SLC3A1*(−0.0750391308399826) + SLC25A37 × (0.103682343486892) + SLC5A6 × (0.289873130489491) + SLCO5A1 × (1.75616206793574) + SCP2 × (−0.0382914831217994). Patients in both the training and internal validation cohorts were separated into high- and low-risk groups according to their median risk values (Figs. 2C and 2D). The relationships between the survival status and risk score of patients in the two cohorts are shown in Figs. 2E and 2F. The expression levels of the eight model genes in the training cohort and internal validation cohort were displayed in the form of heatmaps (Figs. 2G–2H).

### Evaluation of prognostic signature

**FIGURE 3:**
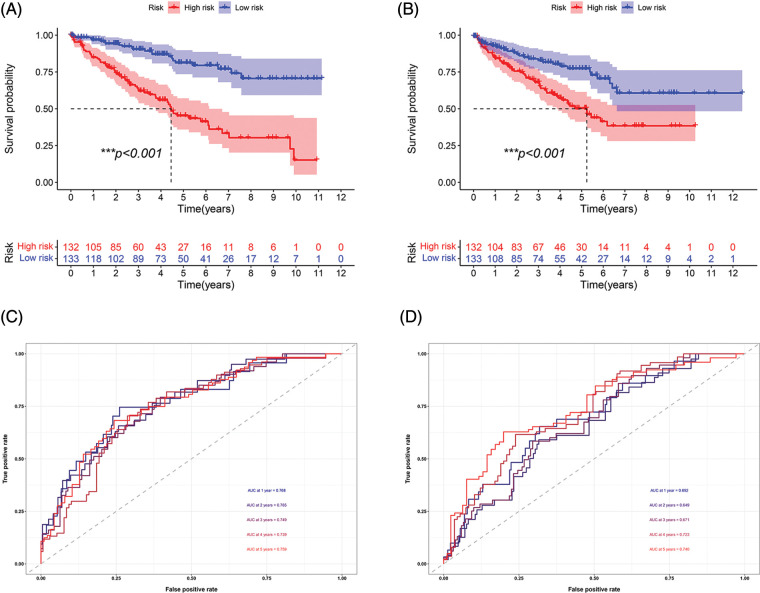
Prognosis and accuracy evaluation of the signature. (A) Survival analysis in the training cohort. Patients in high-risk group had a worse prognosis. (B) Survival analysis in the internal validation cohort. Patients in the high-risk group had a significantly worse prognosis. (C)ROC curve of training cohort. The AUC values in 1, 2, 3, 4, and 5 years of the signature were 0.768, 0.765, 0.749, 0.739, and 0.759, respectively. (D) The AUC values of internal validation cohort in 1, 2, 3, 4, and 5 years were 0.692, 0.649, 0.671, 0.723, 0.740, respectively (***p < 0.001).

**FIGURE 4:**
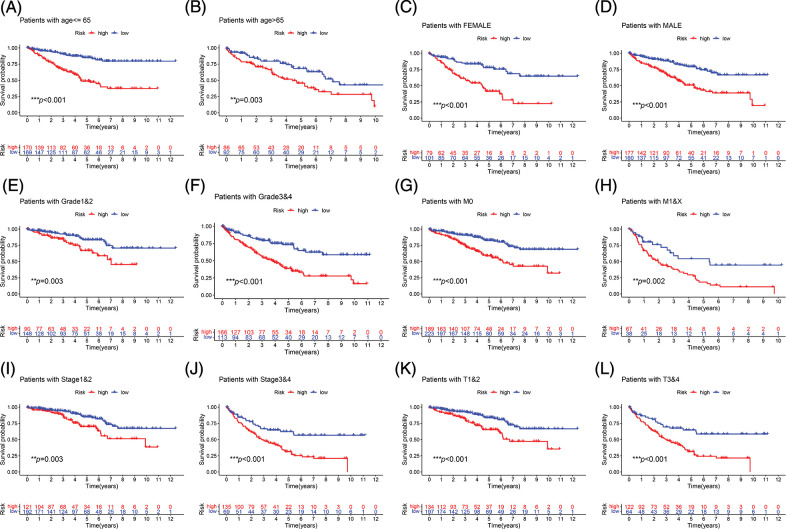
Subgroup survival analysis. (A, B) First, the high-risk group continued to have a worse prognosis in different age groups. (C, D) The high-risk score was also a risk factor for poor prognosis in ccRCC patients in different gender groups. (E–L) Among patients with different grades, M stage, total stage, and T stage, the high-risk group of ccRCC patients continued to have a worse prognosis (**p < 0.01, ***p < 0.001).

Survival analysis was performed to explore the prognostic value of this signature in both cohorts. In the two cohorts, the prognosis of high-risk patients was significantly worse than that of low-risk patients (Figs. 3A and 3B; *p* < 0.001). The ROC curve for the training cohort revealed that the AUC values in 1–5 years of the signature were 0.768, 0.765, 0.749, 0.739, and 0.759, respectively (Fig. 3C). The AUC values for the internal validation cohort at 1–5 years were 0.692, 0.649, 0.671, 0.723, and 0.740, respectively (Fig. 3D). We conducted subgroup analysis to explore the significance of the signature in patients with various clinical features. The high-risk group showed a poor prognosis among different age groups (Figs. 4A and 4B; *p* < 0.01). A high-risk value also contributed to poor prognosis in patients with ccRCC of different sexes groups (Figs. 4C and 4D; *p* < 0.001). Among patients with different M stage, total stage, and T stage, the high-risk group of patients with ccRCC continued to show a worse prognosis (Figs. 4E–4L; *p* < 0.01).

### Independent prognostic significance of the signature

**FIGURE 5:**
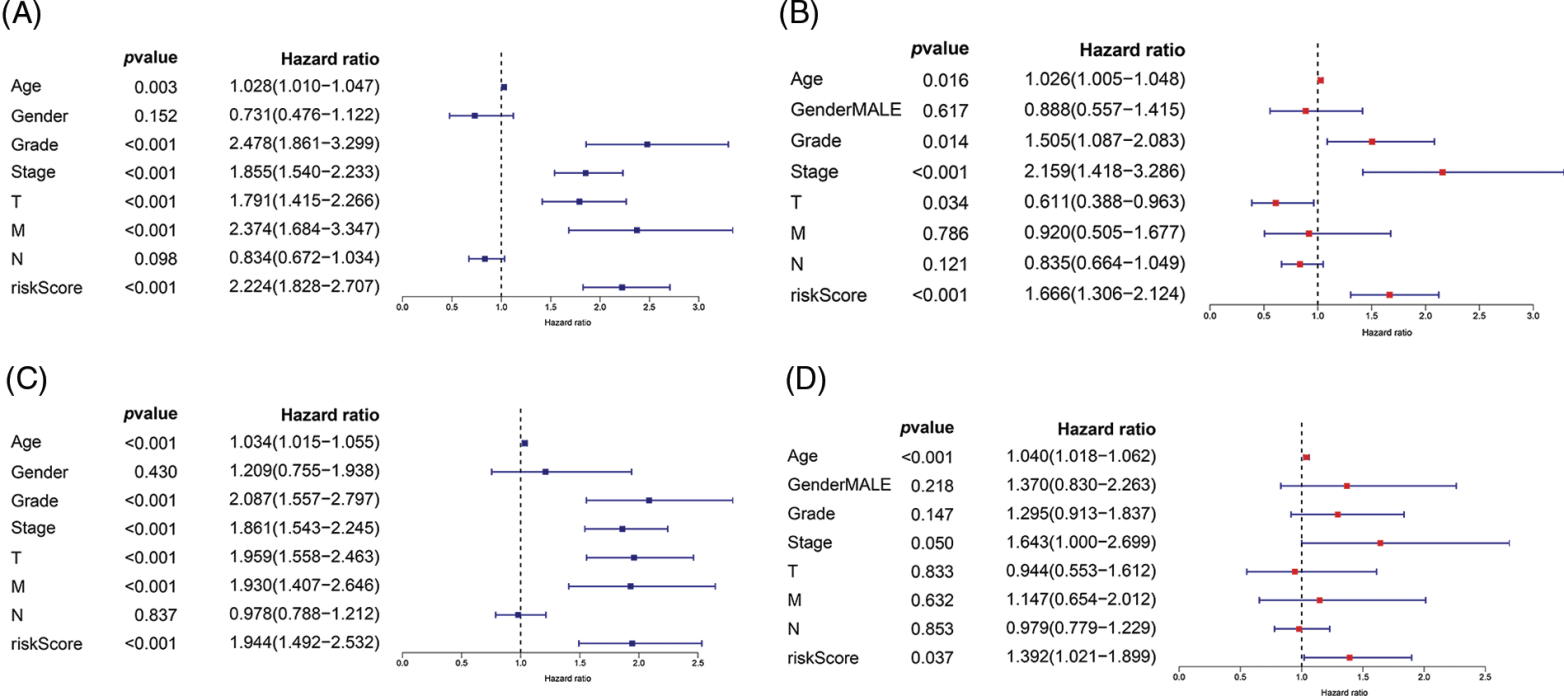
Univariate Cox regression and multivariate Cox regression were used to explore the independent prognostic value of risk score. (A) Univariate Cox regression for training cohort. (B) Multivariate Cox regression for training cohort. (C) Univariate Cox regression for internal validation cohort. (D) Multivariate Cox regression for internal validation cohort.

Univariate and multivariate Cox regression analyses were performed to investigate whether our risk values and other clinical features were independent prognostic indicators of ccRCC. In the training cohort, univariate Cox regression analysis showed that age (HR = 1.028, *p* = 0.003), grade (HR = 2.478, *p* < 0.001), disease stage (HR = 1.855, *p* < 0.001), T stage (HR = 1.791, *p* < 0.001), M stage (HR = 2.374, *p* < 0.001), and the risk score (HR = 2.224, *p* < 0.001) were independent prognostic factors for ccRCC (Fig. 5A). Multivariate Cox regression analysis revealed that grade (HR = 1.505, *p* = 0.014), age (HR = 1.026, *p* = 0.0160), disease stage (HR = 2.159, *p* < 0.001), T stage (HR = 0.611, *p* = 0.034), and the risk value (HR = 1.666, *p* < 0.001) were independent prognostic factors for ccRCC (Fig. 5B). In the internal validation cohort, univariate Cox regression revealed that grade (HR = 2.08, *p* < 0.001), age (HR = 1.034, *p* < 0.001), disease stage (HR = 1.861, *p* < 0.001), T stage (HR = 1.959, *p* < 0.001), M stage (HR = 1.930, *p* < 0.001), and the risk value (HR = 1.944, *p* < 0.001) were independent prognostic factors for ccRCC (Fig. 5C). Multivariate Cox regression revealed that stage (HR = 1.643, *p* = 0.05), age (HR = 1.040, *p* < 0.001), and the risk value (HR = 1.392, *p* = 0.037) were independent prognostic factors for ccRCC (Fig. 5D).

### Analysis of immune correlation

**FIGURE 6:**
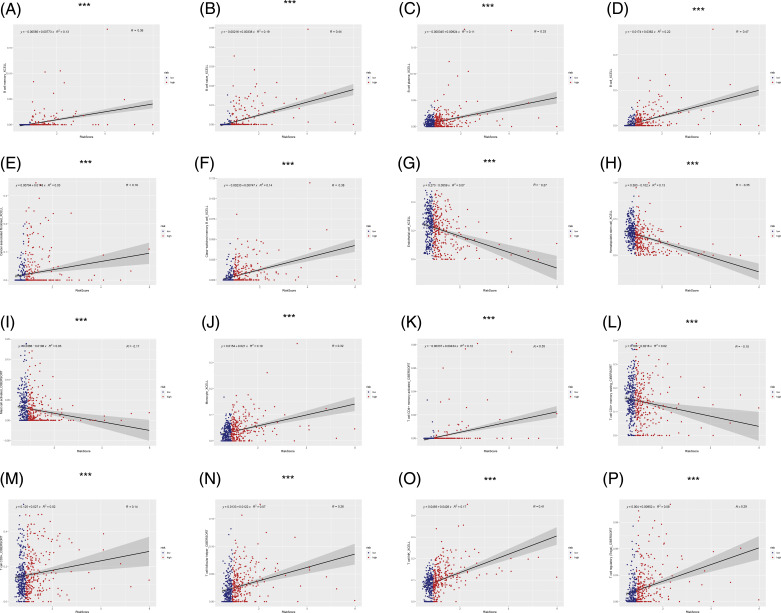
Immunocorrelation analysis. (A–P) B cell memory, B cell naive, B cell plasma, B cell, cancer associated fibroblast, class-switched memory B cell, endothelial cell, hematopoietic stem cell, mast cell activated, Monocyte, T cell CD4+ memory activated, T cell CD4+ memory resting, T cell CD8+, T cell follicular helper, T cell NK, T cell regulatory (Tregs) were significantly related to risk score (***p < 0.001).

The immune microenvironment plays an important role in tumor progression Immunocorrelation analysis showed that activated mast cells, B cells, cancer-associated fibroblasts, endothelial cells, hematopoietic stem cells, monocytes, T cells, and NK cells were significantly related to the risk value (*p* < 0.001, Fig. 6).

### Drug sensitivity analysis

**Figure 7:**
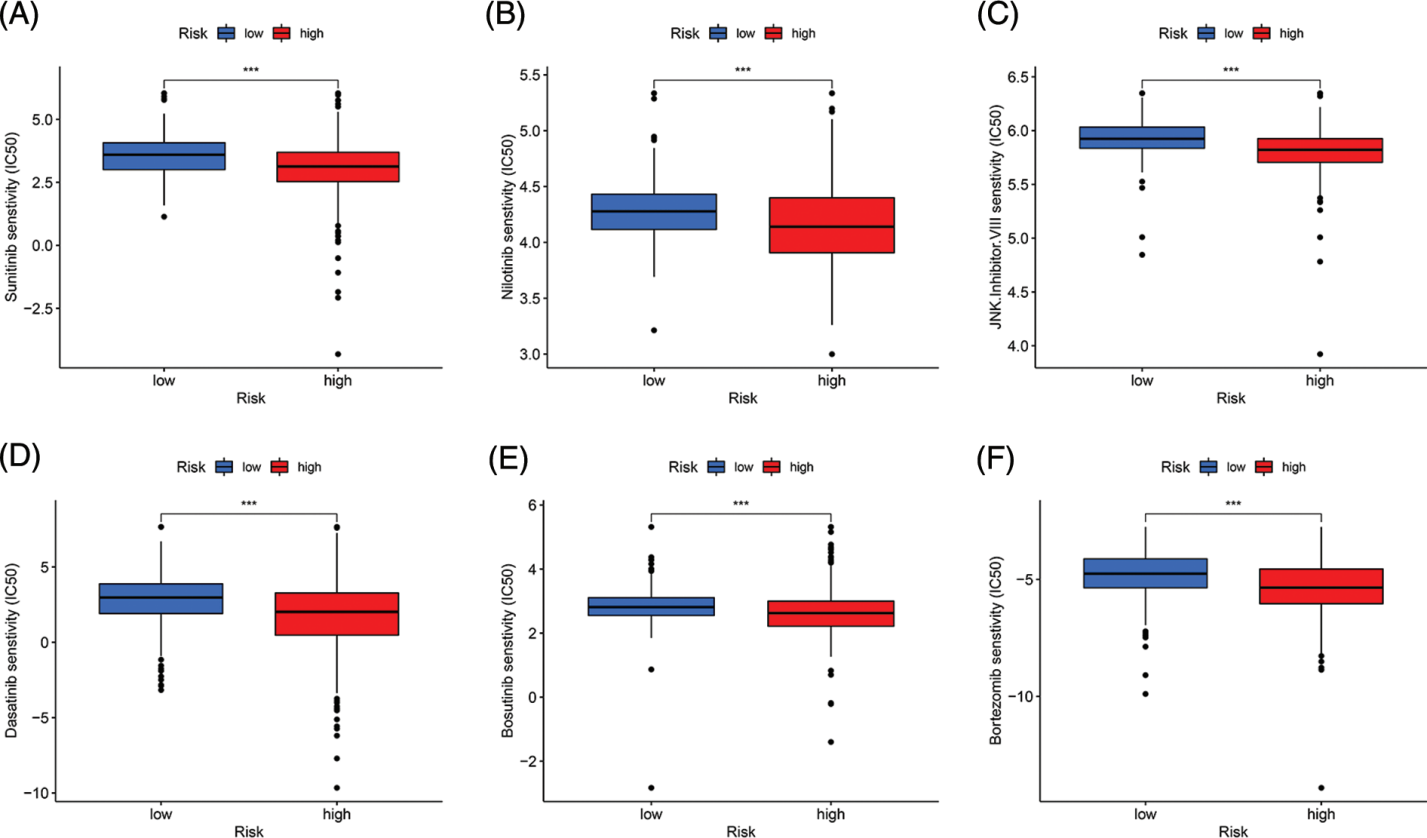
Drug sensitivity analysis. (A–F) Sunitinib, Nilotinib, JNK, inhibitor. VIII, Dasatinib, Bosutinib, and Bortezomib had lower IC50 in the high-risk group (***p < 0.001).

We conducted drug sensitivity analysis to identify more sensitive drugs for targeted treatment of ccRCC. The results showed that sunitinib, nilotinib, JNK inhibitor VIII, dasatinib, bosutinib, and bortezomib had lower 50% inhibitory values in the high-risk group than in the low-risk group, indicating that these drugs may be effective for treating ccRCC (*p* < 0.001, Fig. 7).

### Immune landscape and immune checkpoint analysis

**Figure 8:**
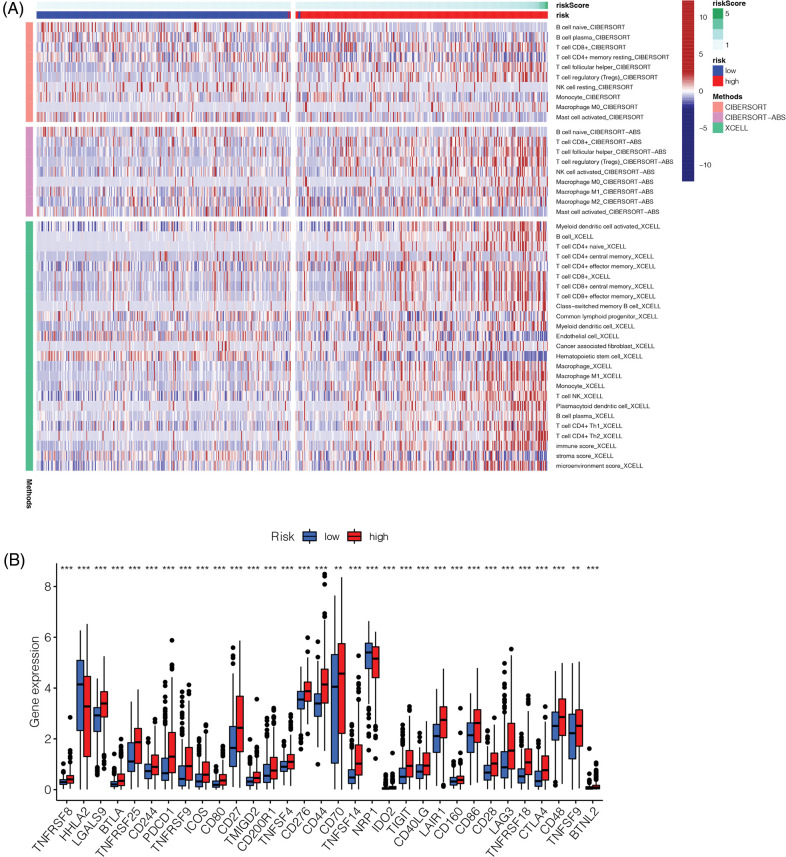
Immune landscape and immune checkpoints analysis. (A) Immune landscape of high-risk and low-risk groups. (B) Analysis of immune checkpoint related genes. Most of the immune checkpoint related genes were up-regulated in the high-risk group (**p < 0.01, ***p < 0.001).

We determined the immune landscape to explore divergence in the immune microenvironments between the high- and low-risk groups to guide immunotherapy (Fig. 8). The high-risk group exhibited higher levels of immune cell infiltration (Fig. 8A). Analysis of immune checkpoint-related genes showed that most immune checkpoint-related genes in the high-risk group were upregulated, indicating that this group may benefit more from blockade of immune checkpoints (Fig. 8B).

### Nomogram construction

**FIGURE 9:**
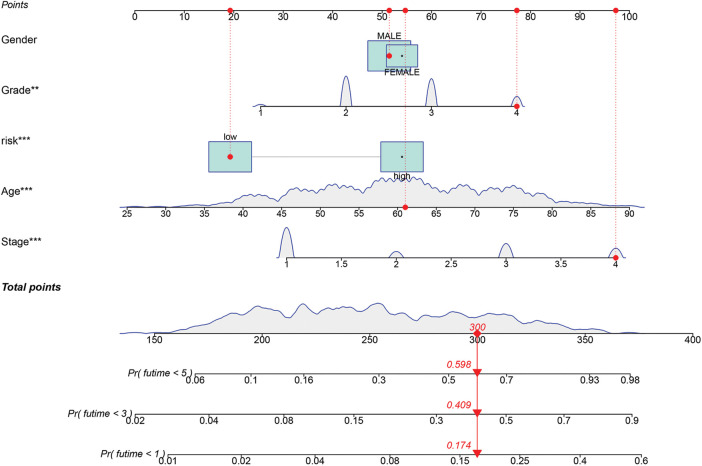
Construction of a nomogram based on risk score. (**p < 0.01, ***p < 0.001).

We constructed a nomogram containing risk scores to further utilize our prognostic model to assess ccRCC prognosis. We randomly selected a patient with ccRCC. The 1-, 3-, and 5-year survival probability determined using the prognostic model were 0.174, 0.409, and 0.598, respectively (Fig. 9).

### External validation of the signature

**FIGURE 10:**
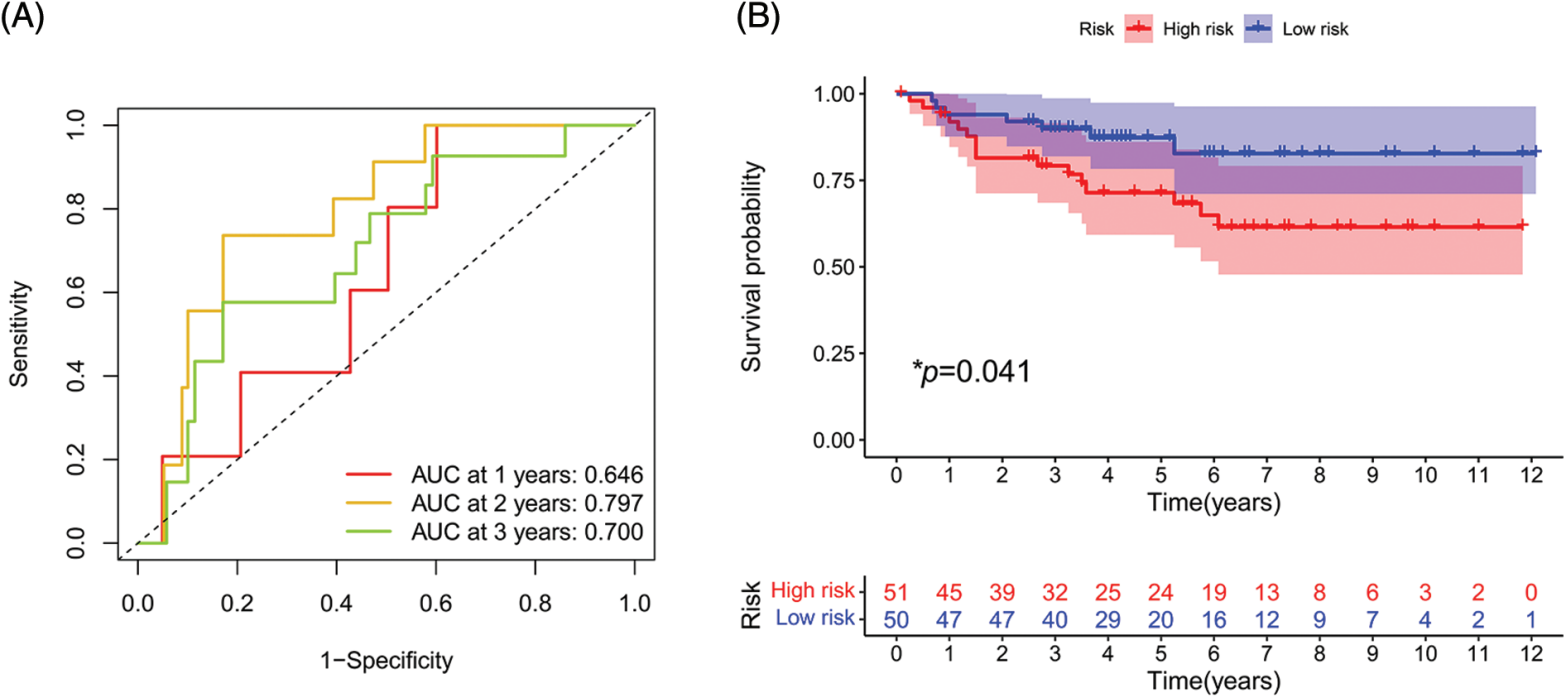
External validation of the signature. (A) ROC curve of E-MTAB-1980 cohort. (B) Survival analysis in E-MTAB-1980 cohort. (*p < 0.05)

To further evaluate the validity and applicability of our signature, we performed external validation using the E-MTAB-1980 cohort. In the ROC curve, the AUCs at 1, 2, and 3 years were 0.646, 0.797, and 0.700, respectively (Fig. 10A). The risk score of each patient was calculated using the risk formula. The cohort was divided into high- and low-risk groups; the survival status of the high-risk group was significantly worse than that of the low-risk group (*p* = 0.041, Fig. 10B).

## Discussion

We performed a comprehensive bioinformatics analysis to investigate the roles of SLC-related genes in ccRCC. First, we constructed a prognostic signature for SLCs in ccRCC, including *SLC25A23*, *SLC25A42*, *SLC5A1*, *SLC3A1*, *SLC25A37*, *SLC5A6*, *SLCO5A1*, and *SCP2*. Our signature also showed good diagnostic accuracy and was an independent prognostic indicator for patients with ccRCC. Immune correlation analysis revealed a notable correlation between various immune cells and the risk score, which may be important in the immune microenvironment. Drug sensitivity analysis identified several drugs that may be useful for treating the high-risk group, which may contribute to the treatment stratification of patients with ccRCC.

Renal cell carcinoma, the most common malignant tumor of the renal system, causes approximately 150,000 deaths worldwide annually, and its incidence is steadily increasing [25–27]. The most common subtype is ccRCC, which accounts for approximately 85% of cases of renal cell carcinoma [28]. Early ccRCC has a good prognosis; however, once progression or metastasis occurs, the prognosis is poor, with a 5-year survival rate of less than 15% [29]. Several prognostic signatures of ccRCC have been identified. Yin et al. constructed a seven-gene signature to stratify patients with ccRCC and evaluate their immunotherapeutic response [30]. They discovered that the high-risk group had an immunosuppressive phenotype with worse prognosis, whereas the low-risk group showed a better response to pD-1 therapy [30]. Chang et al. built a signature containing 11 ferroptosis-related genes to assess the prognosis of patients with ccRCC, in which patients with high-risk values had a significantly poorer prognosis than those with low-risk values [31]. Zhang et al. [32] built a signature of 17 metastasis-related genes using single-cell sequencing to assess the immune response and prognosis of patients with ccRCC. Similarly, high-risk patients showed a worse prognoses. However, SLC-related signatures have not been developed for ccRCC. Our SLC-related signatures has clinical application value in ccRCC.

The eight genes in our model were preliminarily shown to be involved in the pathogenesis and progression of different disease. SLC25A23 is a mitochondrial calcium channel that mediates cell death by inducing oxidative stress [33]. Kim et al. [34] found that SLC25A23 was associated with the prognosis of diffuse large B-cell lymphoma. SLC25A42 is the main CoA transporter in mitochondria [35]. Zhao et al. [36]constructed a 20-gene signature to assess the prognosis of lung adenocarcinoma, in which SLC25A42 is a crucial molecule. SLC5A1 is a sodium-sugar co-transporter [37]. Gao et al. [37] found that SLC5A1 promotes the proliferation and invasion of pancreatic cancer cells through the AMPL/mTOR pathway. SLC3A1 mediates transmembrane transport of cysteine [38], and Jiang et al. [38] found that SLC3A1 promoted breast cancer progression. SLC25A37 is mainly expressed in the mitochondrial intima and mediates iron ion transport [39]. Li et al. [39] found that SLC25A37 is a poor prognostic indicator in pancreatic cancer. SLC5A6 is an Na^+^-dependent multivitamin transporter. Sun et al. [40] found that SLC5A6 is a prognostic marker of gastric cancer. SLCO5A1 is a peptide transporter [41]. Tang et al. [41] suggested that SLCO5A1 is associated with prostate cancer progression. SCP2 mediates the transmembrane transport of cholesterol [42]. Ding et al. found that SCP2 expression was linked to the progression of pituitary adenomas [42]. We combined these eight genes to build a prognostic model to improve the understanding of cancer metabolic reprogramming. Moreover, we showed that compared to patients in the low-risk group, those in the high-risk group were more likely to express immune checkpoint genes, suggesting that these patients may benefit more from immunotherapy.

Although new therapeutic schemes such as vascular and immune checkpoint inhibitors have achieved preliminary benefits in ccRCC, significant drug resistance persists in many patients, leading to treatment failure [43]. Resistance to anticancer therapies can be divided into intrinsic (primary) resistance and acquired (secondary) resistance [44]. However, the mechanisms underlying resistance in ccRCC remain unclear. Several studies confirmed that certain drug transporters in cancer cell membranes play essential roles in the development of drug resistance [43]. Although SLC and ATP cassette transporters are important mediators of multidrug resistance [44], their specific functions and mechanisms of action in ccRCC remain unknown.

We identified high-risk patients as being more sensitive to sunitinib, nilotinib, JNK-inhibitor-VIII, dasatinib, bosutinib, and bortezomib compared to the sensitivity of low-risk patients to these drugs. Sunitinib is a first-line treatment for ccRCC and an inhibitor of tyrosine kinases [45]. Although good initial results have been achieved in many ccRCCs, some patients respond poorly to treatment or develop drug resistance [46]. Therefore, it is necessary to stratify ccRCC sensitivity to sunitinib. Our study revealed that high-risk patients were more sensitive than low-risk patients to sunitinib, which is beneficial for precise treatment of ccRCCs and provides a reference for the application of several other drugs for ccRCC.

However, this study had some limitations. For example, *in vivo* and *in vitro* experiments are needed to validate our results. Furthermore, additional external cohorts and samples are required to reduce bias.

## Conclusions

We developed a prognostic classification method for patients with ccRCC based on *SLC25A23*, *SLC25A42*, *SLC5A1*, *SLC3A1*, *SLC25A37*, *SLC5A6*, *SLCO5A1*, and *SCP2*. This signature allows for ccRCC risk assessment and prognostic classification, in which patients with high-risk ccRCC tend to have a worse prognosis. Our findings can also be used to better understand the interactions between cancer genomes and metabolomics. *In vivo* and *in vitro* investigations are needed to verify our findings.

## Supplementary Materials

SUPPLEMENTARY TABLE 1 Clinical data downloaded from TCGA

SUPPLEMENTARY TABLE 2 41 genes accessed by univariate cox analysis

## Data Availability

AII data involved in this research could be accessed in the TCGA (https://portal.gdc.cancer.gov/), Genecard (https://www.genecards.org/) and Arrayexpress (https://www.ebi.ac.uk/arrayexpress/) databases.
